# Detecting immigrants in a highly genetically homogeneous spiny lobster population (*Palinurus elephas*) in the northwest Mediterranean Sea

**DOI:** 10.1002/ece3.349

**Published:** 2012-08-28

**Authors:** Hamdi Elphie, Goñi Raquel, Dìaz David, Planes Serge

**Affiliations:** 1 USR 3278 CNRS – EPHE, Centre de Biologie et d'Ecologie Tropicale et Méditerranéenne, Université de Perpignan52 Av. Paul Alduy, 66860, Perpignan Cedex, France; 2 IEO – Centro Oceanográfico de BalearesMuelle de Poniente s/n, 07015, Palma de Mallorca, Spain

**Keywords:** Connectivity, early benthic juveniles, microsatellite, *Palinurus elephas*, population structure, spatial structure.

## Abstract

We investigated the genetic structure of early benthic juveniles of the spiny lobster *Palinurus elephas* in the northwest Mediterranean Sea by means of ten polymorphic microsatellite markers. Non-metric Multidimensional Scaling coupled with assignment tests were used as a new approach to further delimit a reference population inside a genetically homogeneous pool of individuals and test for the presence of long distance immigrants. From this approach, we found that most early benthic juveniles collected while settling in the northwest Mediterranean Sea originated from a common larval pool. However, 4.2% of the individuals were classified as immigrants from other genetically differentiated populations, with more immigrants in the south than in the north of the sampled basin. Given currents in the northwest Mediterranean Sea and the long pelagic larval phase of *P. elephas* that lasts several months, this result suggest a restricted homogenized zone in the studied basin with some individuals probably coming from more differentiated populations through the Almeria-Oran Front or the Strait of Sicily.

## Introduction

Connectivity between populations is a key process in understanding marine life cycles and has become a central issue in the fields of conservation and management of marine wildlife. In the marine world, connectivity is mainly determined by dispersal processes throughout oceanic currents and water masses movements. For most benthic fish and invertebrates, whereas the adult stages are generally sedentary or exhibit limited mobility, the larval phase is planktonic, potentially highly dispersive and therefore playing a key role in population connectivity (Grantham et al. 2003). Larval dispersion is shaped by many environmental and behavioral factors that influence the distance and the strength of settlement processes. For a long time, oceans were viewed as open areas without obvious barriers where the circulation of individuals was unrestricted (Strathmann 1990). Thus, marine populations were often considered as highly connected with potential of important gene flow over large geographic distances that could prevent genetic local differentiation. Nowadays and thanks to the improvement of genetic approaches, many studies are showing that the marine realm is more structured than previously thought and that genetic differentiation exists sometimes even over short distances (Todd 1998; Swearer et al. 2002; Hey 2006; Galarza et al. 2009; Hellberg 2009; Casabianca et al. 2011).

Although progress has been made in understanding the processes driving adult and juvenile stages, larval dispersion remains unresolved for most marine populations (Cowen et al. 2007). This lack of knowledge is a fundamental obstacle in understanding population dynamics of marine organisms and furthermore in establishing adapted management plans involving marine protected areas (Sale et al. 2005). In that context, population genetics is the most frequently used approach to investigate larval dispersal and genetic connectivity (Jones et al. 2009). Numerous molecular markers are suitable for these types of studies (Nielsen et al. 2009) among which microsatellites have been largely developed recently because of their high polymorphism (Tautz 1989). Such a high variability contribute revealing fine population differences at small scales and makes microsatellites as effective molecular markers to detect genetic differences even through limited geographic scales (Carreras-Carbonell et al. 2007).

As their definition by Wright (#b^101^), population structure has been widely described using the F-statistic approach (Holsinger and Weir 2009) and this approach is still used and useful despite being based on many assumptions often difficult to validate in natural populations (infinite population, Hardy–Weinberg equilibrium, random mating, etc.) (Guillot et al. 2009). Recent improvements of statistics using Bayesian approaches in the field of population genetics overtake most problems encountered with F-statistics. Particularly, in the field of connectivity, Bayesian methods prove to be powerful for assigning individuals to populations on the basis of multilocus genotypes (assignment methods) (Waples and Gaggiotti 2006; Saenz-Agudelo et al. 2009; Sillanpää 2011). These approaches were able to resolve complex population structuring for further evolutionary, ecological, and conservation studies (Faubet et al. 2007; O'Hara et al. 2008).

The European spiny lobster *Palinurus elephas* (Fabricius, 1787) is an economically important species inhabiting a wide geographic range that extends from the northeast Atlantic to the eastern Mediterranean. The overfished status of its populations is attributed to changes in fishing practices together with growing fishing effort in recent decades (Goñi and Latrouite 2005). The biological characteristics of the species such as a low growth rate, a long life-span jointly to a three to five time lower fecundity than other Palinuridae contribute to the fragility of the stocks (Goñi and Latrouite 2005). The persistence of widely distributed viable populations is likely related to its complex life cycle, with a benthic adult phase of limited mobility (<5 km) and an extensive dispersive larval phase, from 5 to 6 months in Mediterranean to 10–12 months in the Atlantic (Goñi and Latrouite 2005), that potentially allows repopulation from wide open sources. Considering the economic and ecological importance of *P. Elephas*, it is essential to understand gene flow range in order to identify the limits of populations as an important step toward a proper management of this threatened species. For a long time, related research has focused on the ecology of early benthic juveniles (Diaz et al. 2001; Díaz 2010) and our understanding of larval dispersal patterns and population structure is still limited. For long, because of its long larval phase, it has been hypothesized that *P. elephas* have high dispersal ability, and thus high levels of gene flow and low or absence of population structuring at large spatial scales. However, some recent studies have investigated population structure and phylogeography of *P. elephas* at large geographic scales based on adult collections (Groeneveld et al. 2007; Patarnello et al. 2007; Palero et al. 2008, 2009; Babbucci et al. 2010; Palero et al. 2011) and they show that the hypothesis of wide panmixia is now questionable and larval dispersal pattern certainly not resolved. In fact, the emerging picture suggests a barrier of gene flow in the Almeria-Oran Front defining two distinct gene pools generally associated with Atlantic and Mediterranean basins with borders not really defined. Palero et al. (2011) show that Atlantic and Mediterranean populations form two partially overlapping groups and Babbucci et al. (2010) that Azores and Portugal populations seem closely related to northwest Mediterranean population. Furthermore, there are also evidences of another barrier in the Strait of Sicily leading to an east/west differentiation inside the Mediterranean Sea. The easternmost population (Greece) appears well-separated from other Mediterranean samples. These results might indicate, at a large scale, a limited exchange between eastern and western Mediterranean populations as proposed for other marine species, as well as little connection occurring between Atlantic and Mediterranean Sea (Patarnello et al. 2007).

The aim of the present project is to improve the understanding of genetic connectivity and source-sink dynamics for the spiny lobster *Palinurus elephas* at a smaller scale (northwest Mediterranean basin). In such perspective, we focus, for the first time, on collections of recent post-puerulus individuals to investigate, for the first time in that species, patchiness in the recruitment and connectivity pattern. Investigating post-puerulus reduces the variance in genetic differentiation due to cumulating adults of multiple years of recruitment in collections and therefore various network of connectivity from year to year. Using ten microsatellite markers previously developed by Palero and Pascual (2008), our objectives are to determine (1) if there is genetic structuring at regional (100 of Km) and local (10 of Km) spatial scale and (2) what are the exchange patterns among populations.

## Materials and Methods

### Sampling

**Figure 1:**
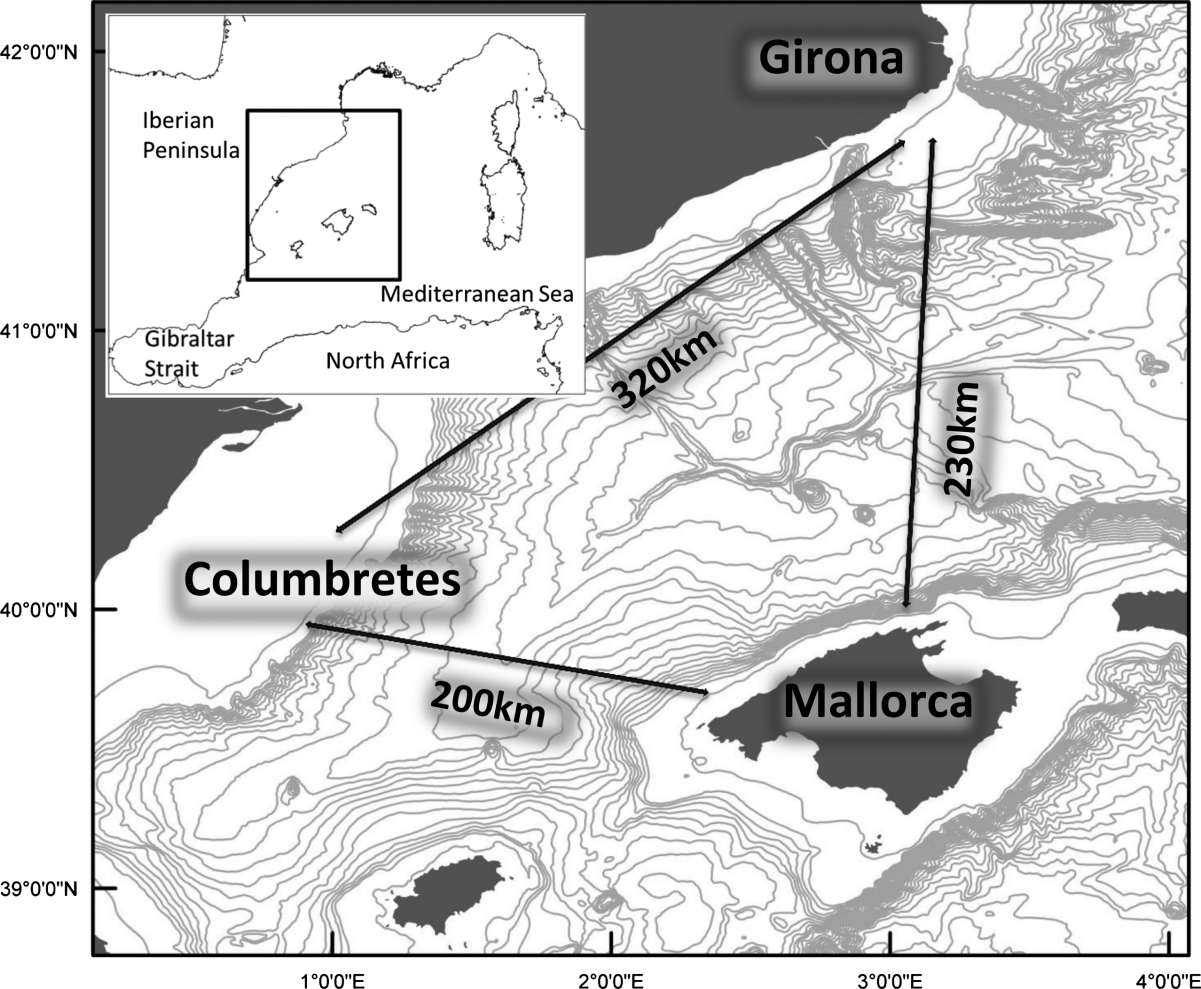
Studied area and geographic position of the three sampled regions.

Sampling was conducted throughout the settlement season of 2010 (June–September 2010) by the Centro Oceanográfico de Baleares of the InstitutoEspañol de Oceanografía (COB-IEO). Three regions within the northwest Mediterranean Sea, separated by more than 200 km (Girona, Columbretes, and Mallorca) were selected and within each region, four locations separated by approximately 10 km were sampled (Fig. 1). In total, 238 early benthic juvenile lobsters (post-puerulus < 18mm CL) were collected from diving surveys. Leg tissues were sampled and stored in 96% ethanol for subsequent DNA extraction.

### DNA extraction and genotyping

DNA was extracted from a total of 238 sampled recruits following the QIAGEN® DX Universal Tissue Sample DNA Extraction protocol. Fourteen polymorphic microsatellites of 15 previously developed for this species (Palero and Pascual 2008) were amplified in three multiplex polymerase chain reactions (PCR) using fluorescently labeled primers and the QIAGEN® Multiplex PCR reagent kit (QIAGEN S.A.S., Courtaboeuf cedex, France). The PCR products were run in an ABI3730xl sequencer (provided by DNAVision S.A. in Belgium) and we sized alleles using GeneMapper version 3.7 (Applied Biosystems France, Villebon sur Yvette, France).

### Genetic polymorphism

Basic genetic statistics were computed in order to validate assumptions required for population genetic analysis such as allele frequency and diversity indexes for the different microsatellites in GenAlEx version 6.4 (Peakall and Smouse 2006). Tests for Hardy–Weinberg deviation and linkage disequilibrium were conducted using GenePop version 4.1.0 (Raymond and Rousset 1995) and sequential Bonferonni corrections were applied. The presence of null alleles was checked using Micro-checker version 2.2.3 (Van Oosterhout et al. 2004).

### Population structure

Population structure was assessed from two different approaches based on different a priori assumptions. First, we used *F*-statistics via an analysis of molecular variance (AMOVA) performed in Arlequin version 3.5.1.2 (Excoffier and Lischer 2010). This analysis partitions the observed genetic variance into components associated with different hierarchical levels. It estimates and tests the percentage of genetic variation explained by differences among regions, within regions, among samples, and within samples. This approach presupposes that sampling locations provide some structure to be tested.

Second, we performed two Bayesian analyses. These two methods differed in the question addressed as one focuses on segregating populations while the other focuses on segregating divergent individuals. The first one was performed using STRUCTURE version 2.3.3 (Pritchard et al. 2000) to detect genetically homogeneous groups of individuals without any a priori information of the spatial distribution of samples. The concept of this approach is to build groups of individuals in order to minimize intra-group variance and maximize inter-group variance by iterative computing. We chose the admixture model and the option of correlated allele frequencies between populations following Falush et al. (2003) considerations. The length of the burn-in period was estimated to be sufficient at 10,000 and the MCMC at 40,000 as proposed by Pritchard et al. (2000). Four runs were carried out for each dataset and the range of possible genetic clusters (K) was set from one to six, which corresponded to the possible number of regions sampled plus three (in case there was substructure within one or more regions). The results were analyzed following Evanno et al. (2005) method using the online version of Dent's Structure Harvester (Earl 2011). This method consists in plotting ΔK (a measure of the mean rate of change of the Log probability for a given K) against K (number of genetic clusters) in order to find the most likely value of K that explains the genetic data. The most likely K is usually viewed on this plot as the presence of a sharp peak (Evanno et al. 2005).

Lastly, we used Primer 6.1.10 (Clarke 1993; Clarke and Gorley 2006) to construct a non-metric multidimensional scaling (nMDS) of pairwise, individual by individual, genetic distances (Smouse and Peakall 1999) between all samples calculated in GenAlEx version 6.4. This analysis gives a two-dimensional graphical representation of the structure based on the genetic distances between individuals without any a priori information of the spatial distribution of samples. Zhu and Yu (2009) have demonstrated the effectiveness of the nMDS method to account for population structure. In the present case, we used the nMDS approach to discriminate outlier individuals and describe genetically homogeneity of the samples.

### Assignment tests

Assignment tests were carried out to assign or exclude individual samples from a given reference population using GeneClass2 (Piry et al. 2004). We used the nMDS procedure to arbitrarily define a reference population. We took individuals within a distance to the barycenter less than or equal to the median as the reference population. This novel approach allows the definition of a reference population based on a genetically homogeneous population. For assigning/excluding the remaining individuals that were not included in the reference population, the Bayesian method of Rannala and Mountain (1997) was chosen. Compared to other likelihood-based and distance-based methods, this method has been described as best adapted in assigning/excluding individuals to populations (Cornuet et al. 1999). Statistical thresholds were estimated by the Monte Carlo resampling algorithm of Paetkau et al. (2004) (*n* = 10,000) implemented in the same software. Piry et al. (2004) have suggested that this algorithm is the most appropriate for this analysis because it takes into account the sample size of the reference population and better reflects the sampling variance associated with the analyzed dataset than other resampling procedures. Individuals were considered immigrants when the probability of being assigned to the reference population was lower than 0.05 (Type I error).

## Results

### Genetic differentiation

The mean expected heterozygosity (0.788 ± 0.032) and the mean observed heterozygosity (0.702 ± 0.037) were high and not statistically different. No evidence of linkage disequilibrium was found between any pair of loci. Four loci (Pael20, Pael53, Pael14, and Pael28) showed deficit of heterozygotes. This deficit is likely to be linked to the presence of null alleles as shown by the results of Micro-checker and therefore we excluded them from further analysis. All 10 remaining loci were considered statistically independent, in Hardy–Weinberg equilibrium and highly polymorphic. Values of observed and expected heterozygosities and number of alleles for the 14 loci are summarized in Table 1.

**Table 1:** Summary of genetic variation data for ten microsatellites loci of *Palinurus elephas* sampled individuals in the northwest Mediterranean Sea from three sampling regions (Columbretes, Girona, and Mallorca). *N*, number of individuals analyzed per region and in parenthesis per location within region; Na, number of alleles; Ho and He, observed and expected heterozygosity, respectively; in pale gray, loci that have been excluded

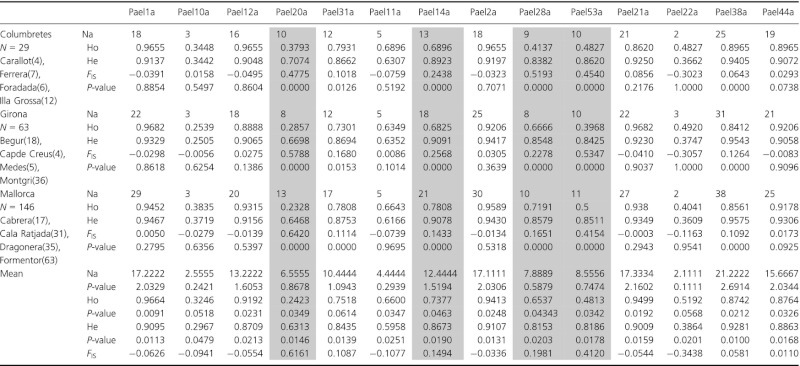

### Population structure

**Figure 2:**
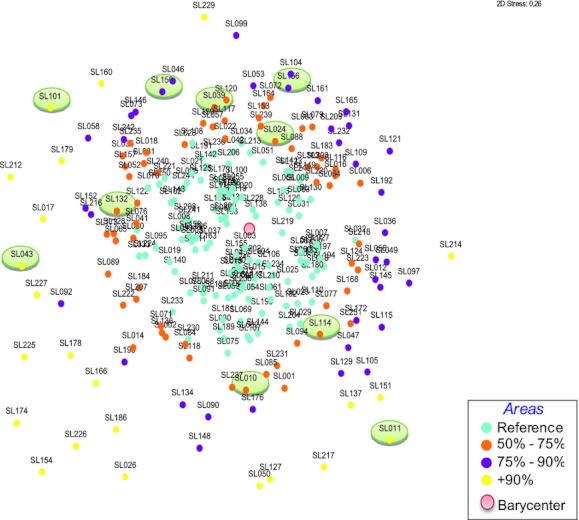
Non-metric multidimensional scaling of *Palinurus elephas* samples from the northwest Mediterranean Sea based on genetic distances among individuals at ten microsatellite loci. Each point corresponds to an individual and each color represents an area constructed according to the Euclidean distance to the barycenter. Circled individuals have been identified as immigrants by assignment analyses.

Based on sampling locations, the AMOVA analysis indicates an absence of genetic structure at both regional and local scales. Overall, within sample variance explained 99.9% of the total variance. The remaining 0.1% of variance explained by among population differences within regions was not significantly different from zero, with an *F*_ST_ equal to 0.0005 (*P*-value = 0.6080). The results of both Bayesian methods (STRUCTURE and nMDS) also confirmed the absence of genetic clustering even while not considering the sample structure. Results from the graphic representation of Evanno's ΔK revealed the presence of a peak at K = 3.0, but when plotting the relative membership of each individual to each of the three clusters, all individuals presented equal probabilities to belong to each one of the three clusters. Finally, results of the nMDS representation (stress = 0.26) also supported the presence of one single homogeneous population with a single cloud of points representing each individuals (Fig. 2).

### Assignment tests

**Figure 3:**
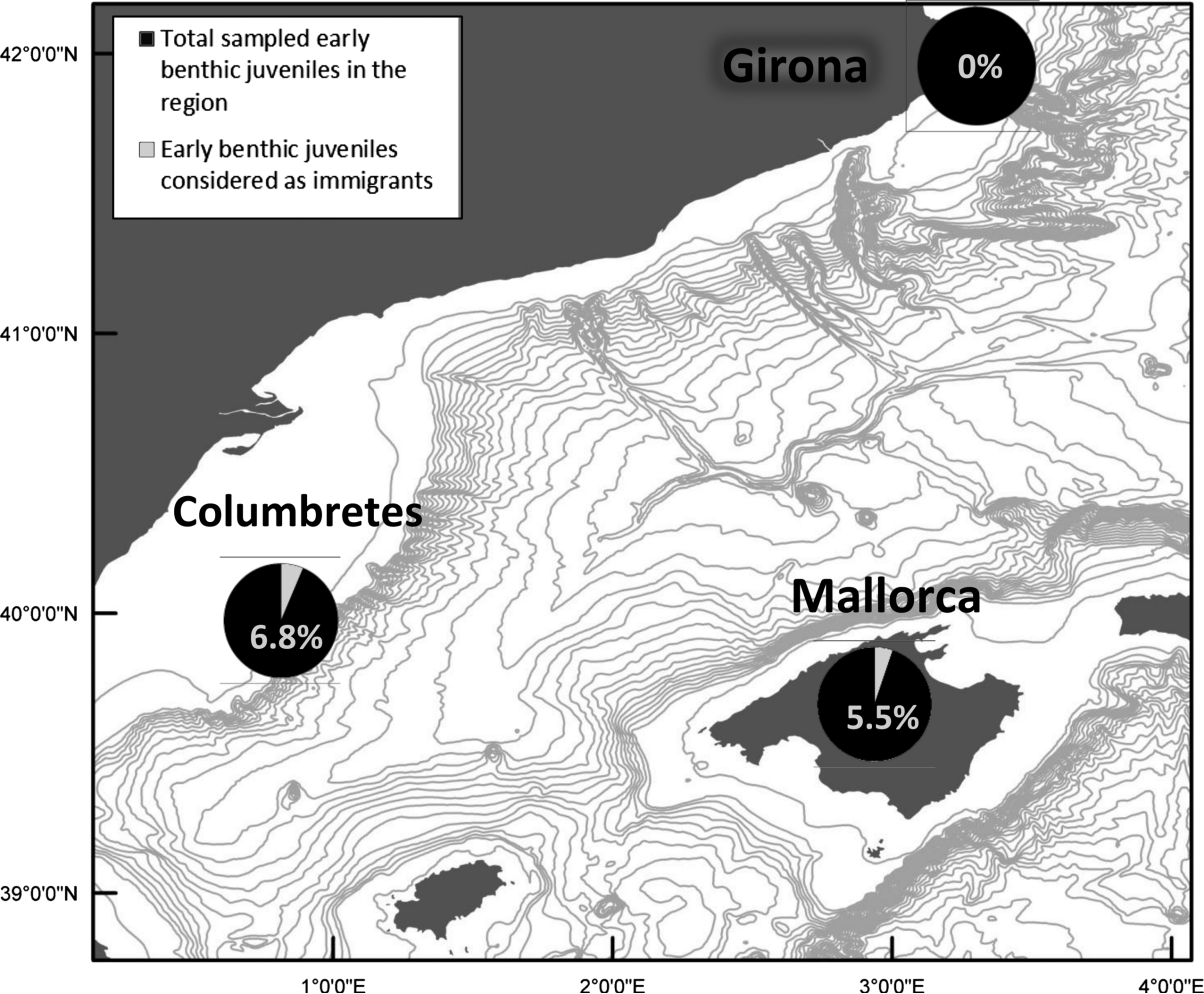
Graphical representation of the proportion of immigrants (%) of *Palinurus elephas* in the three regions sampled in the northwest Mediterranean Sea.

Of the 238 individuals tested, 117 were incorporated in the reference population. From assignment tests and assuming that the reference population comes from a unique gene pool, 10 individuals (4.2%) among the most divergent are distinguished as immigrants (*P* < 0.05), as they are not assigned to the reference population. Three of these immigrants are located within the 10% most peripheral points (to the barycenter) in the nMDS, whereas two immigrants belong to the 75–90% area and five individuals belong to the 50–75% area (Fig. 2). Based on these results, we compared the percentage of immigrants among sampled regions (with a bilateral Fisher test). Immigrants appeared significantly more in Mallorca and Columbretes than in Girona where no immigrants were found (Fig. 3).

## Discussion

The present survey on *Palinurus elephas* highlights an overall genetic homogeneity among locations using both frequency analysis and Bayesian methods. This result fits with previous works on lobster that also found genetic homogeneity at even larger spatial scale (Palero et al. 2008, 2011). However, we notice that while we found no structuring of early benthic juveniles (EBJ) samples, Babbucci et al. (2010) found that Columbretes adults are differentiated from Mallorca and Menorca ones. The different degree of structure found between the two studies is likely to derive from difference in sampling strategy. First, the number of individuals they sampled can be considered low (<25 in each locality) and thus can increase a sampling error (Waples, #b^100^). This limitation in sampling has also been noticed by Palero et al. (2011) regarding differences in results they obtained. Moreover, Babbucci et al. (2010) have sampled adults between 1998 and 2006 while our samples were EBJ of the same settlement season. In fact, recent studies have demonstrated the importance of the temporal scale to understand connectivity and dispersal patterns (Calderón et al. 2009; Anderson et al. 2010; Calderón and Turon 2010; Calderón et al. 2012). In species with a long larval dispersal phase, it is assumed that the genetic composition of settled individuals determines the genetic makeup of populations (Watts et al. 1990), but other studies suggest that forces causing genetic differentiation can act locally and occur in a single generation (Planes and Lenfant 2002; Pujolar et al. 2006). In these cases, the genetic diversity within a cohort can vary from year to year and be different from the overall diversity of adult populations (Calderón and Turon 2010). Indeed, local post-settlement selective forces can cause a reduction of genetic variability within cohorts over time (Planes and Lenfant 2002).

Our results clearly show that the northwest Mediterranean population of *P. elephas* is genetically homogeneous, and thus that the different barriers found in this area for other species (Galarza et al. 2009; Mokhtar-Jamaï et al. 2011; Schunter et al. 2011) did not limit larval dispersal of P. elephas.

However, new in this frame work, the combination of nMDS and assignment tests showed that 4.2% of the EBJ sampled are immigrants with more immigrants in the southern locations (i.e., immigrants are individuals statistically diverging from the reference population). The use of this combination was implemented to overtake different problems given by Bayesian approaches. It has been proven that Bayesian methods are powerful approaches that overtake problems encountered with *F*-statistics (Faubet et al. 2007), particularly in the field of population structure and connectivity. Among several, we can mention Baums et al. (2006) that detected a genetic barrier for a coral species (*Acroporapalmata*) using Bayesian methods, whereas classic frequency analysis approaches were showing homogeneity. However, the main limitation of these approaches is that they are poor predictors of genetic structure and fail to correctly assign individuals to populations when gene flow among populations is moderate or, in our case, high (FST < 0.05) (Cornuet et al. 1999; Waples and Gaggiotti 2006; Saenz-Agudelo et al. 2009). This restriction is mostly due to the fact that these methods rely on differences of allelic frequencies among genetic clusters. For assignment tests in particular, this can lead to bias in defining reference populations. To overtake the problem of reference population, we used an nMDS representation to characterize the reference population and making the characteristics of the reference population independent of sampling. Under a situation of high gene flow, where there is no a priori information, the nMDS allow to define a reference population inside a homogeneous group. This combined approach allows us to go further in the understanding of genetic dynamics in a homogeneous population making possible to test outliers as potential immigrants. In this case, we were able to demonstrate that 4.2% of individuals collected were immigrants.

Given the differentiation of the northwest Mediterranean population compared with other regions, the long larval phase and the adapted morphology of the larvae to be dispersed by currents, immigrants can come from the entire distribution of *P. elephas* following oceanic currents. Two main currents enter in the northwest Mediterranean Sea. The first one, coming from the East, enters through the Corsica Channel (Salat 1996) and can bring some immigrants coming from eastern Mediterranean differentiated populations. The Strait of Sicily that appears as gene barrier for some species (Patarnello et al. 2007) has recently been investigated for the genetic structure of *P. elephas* and seems to play a role in the differentiation of eastern most populations (Palero et al. 2011). The second current is a surface current coming from the Atlantic Ocean through the Strait of Gibraltar (Salat 1996) that enters the northwest Mediterranean Sea and seems to genetically homogenize populations at both sides of the Strait. Studies by Palero et al. (2008), Babbucci et al. (2010) and Palero et al. (2011) dealing with the same species at larger geographic scales suggest that the Atlantic population of *P. elephas* is differentiated from the Mediterranean population, but with populations of Azores and Portugal coast closely related to the northwest Mediterranean populations. The little connectivity found in these studies and the distribution of immigrants documented here both lend support to a limited gene flow of *P. elephas* through the AOF. However, given the lack of outgroups in our study, we cannot determine whether one hypothesis is better than the other. Thanks to the combination of assignment methods and nMDS representation and the geographic pattern found in the repartition of immigrants, we can speculate that individuals coming from more differentiated Atlantic populations disperse into the Mediterranean Sea through the Almeria-Oran Front. Obviously, further analyses, comparing immigrants' genetic signature with populations' genetic signatures in both Atlantic and Mediterranean populations need to be performed to precisely define their origins.
